# Deciphering Atypical Signals Present in Fluorescent In Situ Hybridization Assays in the Diagnosis of Soft Tissue Sarcomas

**DOI:** 10.7759/cureus.86795

**Published:** 2025-06-26

**Authors:** Alexandra B Papakosta, Louisa G Mahaira, Eftimios S Dimitriadis, Helen N Rizou, Anastasios I Kyriazoglou, Kalliopi Stefanaki, Vassilios Papadakis, Antonis Kattamis, Fragiski A Anthouli

**Affiliations:** 1 Genetics, "Agios Savvas" General Anti-cancer and Oncological Hospital of Athens, Athens, GRC; 2 Medical Oncology, Attikon University Hospital, National and Kapodistrian University of Athens, Athens, GRC; 3 Pathology, "Agia Sofia" Children's Hospital, Athens, GRC; 4 Pediatric Hematology-Oncology, "Agia Sofia" Children's Hospital, Athens, GRC; 5 Pediatric Hematology-Oncology, National and Kapodistrian University of Athens School of Medicine, Athens, GRC; 6 Biomedical Sciences, University of West Attica, Athens, GRC

**Keywords:** atypical signals, biomarker, fish, ngs, soft tissue sarcomas

## Abstract

Soft tissue sarcomas (STS) represent a heterogeneous group of rare tumors of mesenchymal origin. This group of solid tumors includes more than 120 different histotypes commonly difficult to be accurately diagnosed. Thus, differential diagnosis of these tumors is of utmost importance for patients’ clinical management and usually includes more than one type of test. Fluorescence in situ hybridization (FISH) is one of the most widely applied methods for the detection of fusion genes that characterize the majority of STS. Our routine diagnostic practice includes FISH assay along with other appropriate molecular tests. Atypical signals have been observed in several cases of FISH assays on STS samples that do not fulfill the established diagnostic criteria for rearrangements. Thus, the aim of this study was to investigate this observation and pinpoint their utility in STS diagnosis.

## Introduction

Balanced or unbalanced chromosome (genomic) rearrangements are recognized as a frequent cause of somatic mutation in neoplasia. Genomic rearrangements emerge when chromosomes break and subsequently rejoin haphazardly. Rearrangements may occur independently or in the course of complex genomic alterations shattering chromosomes [1] or rejoining them in chains or other structures (chromoplexy) [2]. Genomic rearrangements can lead to mutations that drive cancer by various means, one of which is the creation of gene fusions. These fusions usually arise from translocations, which are often reciprocal in nature. However, an exception is the TMPRSS2-ERG fusion found in prostate cancer, which can result from a process known as chromoplexy. Gene fusions that promote cancer are especially frequent in leukemia as well as in tumors of the bone and soft tissues [3].

Gene fusions, chromosomal alterations that bring together two formerly separate coding or regulatory regions, occur in about one-third of all soft tissue sarcoma (STS) cases. Due to the close link between specific gene fusions and particular morphological subtypes of STS, they serve as valuable diagnostic tools [4]. Both the World Health Organization (WHO) and the National Comprehensive Cancer Network (NCCN) endorse the use of gene fusions as supplementary diagnostic markers for STS. Most gene fusions are currently identified by fluorescence in situ hybridization (FISH), immunohistochemistry (IHC), reverse transcription-polymerase chain reaction-based Sanger sequencing, or next-generation sequencing (NGS) techniques.

FISH is a cytogenetic technique that implements fluorescent probes to target and bind specific chromosome regions with Complementary DNA (cDNA) sequences. Developed by biomedical scientists in the 1980s, this method enables the detection and localization of particular DNA sequences. A fluorescence microscope is used to visualize the probes attached to the chromosomes [5]. Today, there is a relatively extensive catalog of diagnostically useful chromosomal translocations and their associated chimeric genes. Commercially available break-apart (BrAp) probes are used for the detection of these translocations, exhibiting high specificity. Dual-color BrAp probes are designed to hybridize to DNA sequences that flank the common breakpoints in a given gene. The part of the probe complementary to the 5’ part of the gene brake point area is labeled with a different fluorescent dye than the 3’ part. The spatial dissociation of dual-color probes, typically labeled with green and orange fluorophores, indicates a genomic rearrangement, thereby suggesting the occurrence of a gene-specific chromosomal translocation. The high degree of specificity of probes generally makes their application and interpretation unambiguous. However, there have been reports of atypical or abnormal interphase FISH results [6].

This study was designed to assess the efficacy of the dual-color BrAp FISH probes for gene fusion testing in STSs. Furthermore, we aimed to investigate whether atypical FISH signals could represent gene translocation and thus acquire diagnostic value. 

## Materials and methods

Tissue samples and pathology

This study was approved by the Ethics Committee of the University of West Attica and the Scientific Advisory Board of the "Agios Savvas" General Anti-cancer and Oncological Hospital of Athens.

Most of the specimens analyzed in the present study are from pediatric patients.

Formalin-fixed, 4 µm thick, paraffin-embedded tissue sections of all specimens were immunostained using the Bond Polymer Refine Kit on the BOND-MAX Autostainer (Leica Biosystems), according to the manufacturer’s standard protocols. All specimens were analyzed using a general IHC sarcoma panel of antibodies, comprising a set of markers including vimentin, alpha-smooth muscle actin (SMA), desmin, myogenin, S100, D34, CD31, c-KIT, caldesmon, neuron-specific enolase (NSE), CD99, FLI1, AE1/AE3, cytokeratin, epithelial membrane antigen (EMA), and transducing-like enhancer of split 1 (TLE1). The immunohistochemical expression was evaluated by two independent pathologists as the percentage of positive tumor cells in the most strongly stained areas, relative to the total number of tumor cells counted. Furthermore, additional stainings with more specific antibodies (e.g., PAX5 and SYT), based on the initial IHC results, were also performed.

Fluorescence in situ hybridization

The ZytoLight SPEC Dual Color BrAp Probe system was used for the qualitative detection of translocations involving each of the genes tested, based on the suspected type of STS, in formalin-fixed, paraffin-embedded (FFPE) specimens, according to the manufacturer’s instructions.

Briefly, representative 2-4 µm thick, FFPE tissue sections were selected by an experienced pathologist for the detection of gene translocations. Sections from normal tissue were used as negative controls. The slides were heated, deparaffinized in fresh xylene, hydrated in ethanol solutions, washed in distilled water, and then incubated in Heat Pretreatment Solution Citric (ZytoLight FISH-Tissue Implementation Kit; ZytoVision, Bremerhaven, Germany). Subsequently, the slides were washed again in distilled water and allowed to air dry. They were then treated with pepsin solution, washed again, dehydrated in graded ethanol solutions, and air-dried. The appropriate FISH probe was applied to the areas of interest and sealed with rubber cement. A co-denaturation step (of probe and target DNA) for 10 minutes was followed by overnight hybridization at 37 °C. After 16 hours of incubation, the slides were washed again in the provided wash solution, dehydrated, counterstained with DAPI/Antifade (ProLong Gold Antifade Reagent with DAPI; Life Technologies), and analyzed.

Analysis was performed using a Zeiss Axioplan fluorescence microscope (Carl Zeiss AG, Oberkochen, Germany) equipped with the appropriate filter combinations and the ISIS digital imaging system and software (MetaSystems, Altlußheim, Germany).

The evaluation of FISH signals followed the criteria below: (i) at least 100 intact cells were scored for the presence of a BrAp signal; (ii) overlapping nuclei were excluded from the analysis; (iii) a probe signal was classified as "split" (break-apart) when the green and orange fluorescence signals were separated by a distance at least twice the diameter of an individual hybridization signal; and (iv) a sample was considered positive for gene translocation if the number of nuclei exhibiting BrAp signals exceeded the threshold established by the control specimen, set at 15%.

To establish the cut-off level for any gene rearrangement, we performed FISH analysis on 10 normal tissues, and 50 nuclei were scored for the presence of BrAp signals. The cut-off value was calculated at 15%. All data presented herein are the results of two specialized independent investigators. 

Molecular tests

Molecular analysis was carried out in the Laboratory of Genetics at the "Agios Savvas" General Anti-cancer and Oncological Hospital of Athens. An expert pathologist selected one paraffin block from each specimen, and approximately two to three sections of 5 µm from surgical specimens or four sections of 10 µm from biopsies were obtained. Total RNA extraction was performed using the Total RNA FFPE XS Mini Kit (Macherey-Nagel).

Depending on the availability of the sample, RT-PCR or NGS was applied for the detection of fusion genes.

Real-time polymerase chain reaction analysis

cDNA was synthesized using approximately 100 ng of total RNA, random hexamers, and SuperScript II Reverse Transcriptase (Invitrogen, Thermo Fisher Scientific), according to the manufacturer's instructions. Real-time polymerase chain reaction (RT-PCR) was performed on this material using primers and probes specifically designed to detect a specific fusion gene in each case (Custom MGB qPCR TaqMan Probes 4316034, Pre-Designed Primers Set 4304970; Thermo Fisher Scientific). The assay was performed on an ABI 7500 instrument using TaqMan Universal PCR Master Mix (Applied Biosystems).

Next-generation sequencing analysis

A sarcoma-specific NGS test was performed on a number of samples. Total RNA isolated from FFPE tissue, as described previously, was reverse transcribed using the VILO system (Thermo Fisher Scientific). Subsequently, libraries were synthesized using a sarcoma fusion panel designed in our laboratory (WG-IAD186419-SARCOMA PANEL, AmpliSeq; Thermo Fisher Scientific), which includes 86 different chimeric transcripts representing the majority of genetic aberrations in sarcomas. Library preparation was performed using the Ion AmpliSeq Library Kit 2.0 (Thermo Fisher Scientific), according to the manufacturer’s instructions. Normalization of NGS data was achieved using the Ion Library Equalizer™ Kit (4482298, Thermo Fisher Scientific), according to the instructions. All libraries, at equal molarity, were analyzed on the Ion S5 GeneStudio System, and results were obtained using Ion Torrent Software (v5.20, Thermo Fisher Scientific).

## Results

Fluorescence in situ hybridization analysis 

In the present study, a total of 135 specimens of STS/PNET (primitive neuroectodermal tumors) were analyzed, as shown in Table 1.

**Table 1:** STS/PNET analyzed in this study FISH, fluorescence in situ hybridization; PCR, polymerase chain reaction; NGS, next-generation sequencing; STS, soft tissue sarcoma; PNET, primitive neuroectodermal tumors; BrAp, break-apart

Gene (BrAp) tested	Cases analyzed by FISH	Cases FISH positive (%)	Cases analyzed by PCR/NGS	Cases PCR/NGS positive (%)
EWSR1	71	48 (67.6)	50	34 (68)
SS18	29	13 (44.8)	17	5 (29.4)
FOXO1	20	2 (10)	16	2 (12.5)
FUS	12	4 (33)	9	2 (22.2)
DDIT3	3	3 (100)	1	1 (100)
Total	135		93	

Most of the samples were diagnosed as Ewing/Ewing-like sarcomas and tested for the presence of the EWSR1 BrAp rearrangement. Out of the 71 specimens tested, 48 were found to harbor the EWSR1 translocation (67.6%). For 50 of these specimens, FISH results (positive or negative) were confirmed by RT-PCR or NGS analysis, as shown in Table 2, whereas for the remaining cases, no tissue was available for molecular testing.

**Table 2:** Ewing/Ewing-like sarcoma cases analyzed in this study Out of 71 specimens tested with the EWSR1 break-apart probe, 4 cases, initially considered negative, exhibited atypical signals. When these cases were tested by PCR or NGS, an EWSR1 fusion gene was detected. It should be noted that molecular testing was not performed in all cases due to limited tissue availability. FISH, fluorescence in situ hybridization; PCR, polymerase chain reaction; NGS, next-generation sequencing

EWSR1 cases	FISH results	Molecular test confirmed
n=48	+	n=34
n=19	-	n=12
n=4	-/atypical	+
Total	71	50

The second most frequent type of mesenchymal tumor among the specimens tested in our laboratory was synovial sarcoma, characterized by the presence of the SS18 translocation. A total of 29 specimens were tested, and 13 were found to harbor the specific translocation (44.8%). FISH results (positive or negative) were confirmed in 17 of these cases by either RT-PCR or NGS analysis, as shown in Table 3. For the remaining cases, no tissue was available for molecular testing.

**Table 3:** Synovial sarcoma cases analyzed in this study Out of 29 specimens tested with the SS18 break-apart probe, only 17 were also analyzed by a molecular test due to insufficient tissue availability. No atypical signals were observed. FISH, fluorescence in situ hybridization

SS18 cases	FISH results	Molecular test confirmed
n=13	+	5
n=16	-	12
Total	29	17

Accordingly, for rhabdomyosarcomas, a total of 20 specimens were tested by FISH and only two were found positive for the FOXO1 translocation (10%, Table 4).

**Table 4:** Rhabdomyosarcoma cases analyzed in this study Out of 20 specimens tested with the FOXO1 break-apart probe, three cases that exhibited atypical signals were initially considered negative. When these were tested by PCR or NGS, a FOXO1 fusion gene was detected. Four specimens were not analyzed by PCR or NGS due to insufficient tissue availability. FISH, fluorescence in situ hybridization; PCR, polymerase chain reaction; NGS, next-generation sequencing

FOXO1 cases	FISH results	Molecular test confirmed
n=2	+	n=2
n=15	-	n=11
n=3	-/atypical	+
Total	20	16

Furthermore, 15 specimens of myxoid liposarcomas were tested either for FUS BrAp or DDIT3 break-apart signals, and seven were found to be positive for these translocations (four cases for FUS BrAp and three for DDIT3 BrAp). For 10 of these cases, the results were confirmed by molecular testing, as shown in Table 5.

**Table 5:** Myxoid liposarcoma cases analyzed in this study Out of 15 specimens tested with either FUS or DDIT3 break-apart probes, 10 were analyzed by PCR or NGS. Due to insufficient tissue availability, five specimens were not tested molecularly. No atypical signals were observed. FISH, fluorescence in situ hybridization; PCR, polymerase chain reaction; NGS, next-generation sequencing

FUS or DDIT3 cases	FISH results	Molecular test confirmed
n=7	+	n=3
n=8	-	n=7
Total	15	10

**Figure 1:**
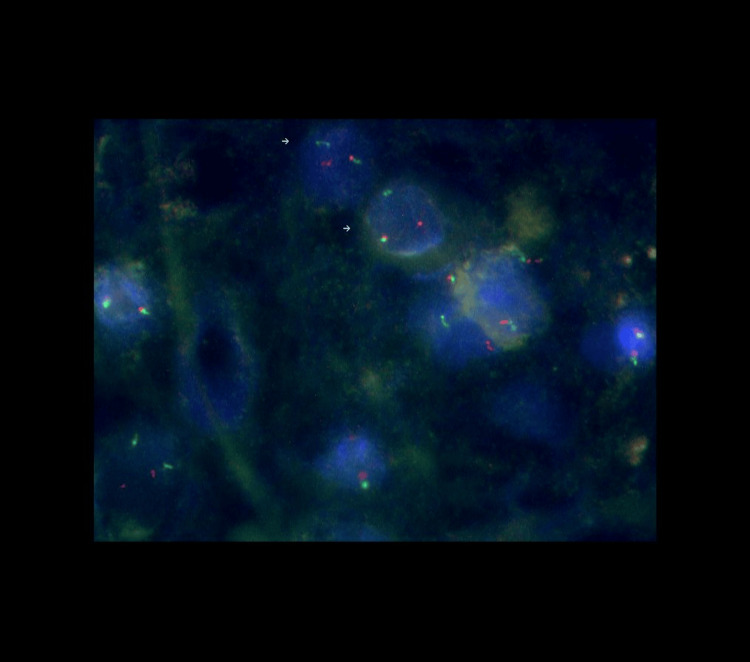
Normal fusion and break-apart signals Paraffin sections were analyzed using a dual-color break-apart probe. With the appropriate filter sets, the hybridization signals appear green (distal to the EWSR1 breakpoint region) and orange (proximal to the EWSR1 breakpoint region). Normal cells harbor two green/orange fusion signals, while aberrant cells harbor one fusion signal, one separate green signal, and one separate orange signal (indicated with small white arrows).

**Figure 2:**
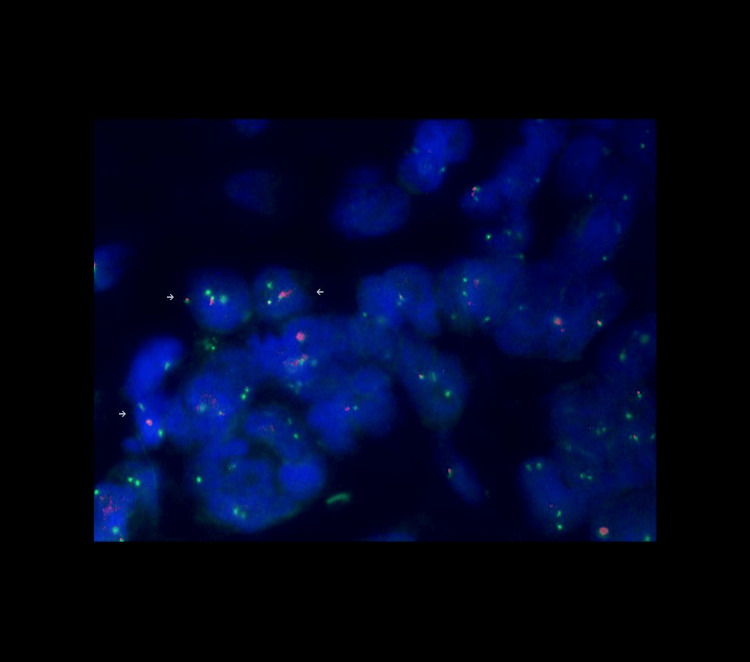
Typical and atypical break-apart signals Paraffin sections were analyzed using a dual-color break-apart probe. With the appropriate filter sets, the hybridization signals appear green (proximal to the FOXO1 breakpoint region) and orange (distal to the FOXO1 breakpoint region). Small white arrows indicate cells with atypical break-apart signals (loss of the orange signal).

An interface of cells harboring normal fusion signals (F) and typical BrAp signals is depicted in Figure 1, whereas atypical signals, consisting of one or more fusion signals and one or more green signals (indicating loss of the corresponding red signal), are shown in Figure 2.

Molecular testing and fluorescence in situ hybridization analysis variance

In our analysis, some discrepancies were observed between FISH results and molecular testing results, which are presented in detail in Table 6. In all these cases, "atypical signals" were observed. This term refers to the detection of one fusion signal along with either one green (1F/1Gr) or one red (1F/1R) signal per nucleus, indicating the loss of a portion of the tested gene. Various combinations of atypical signals were observed across multiple cases. For example, instead of the typical 1F/1R or 1F/1Gr pattern, multiple signals were detected, such as 2F/1Gr, 3F/2Gr, and 2F/1R, 3F/3R. All of these signal patterns are reported in our results as "atypical signals."

**Table 6:** FISH BrAp and molecular test result discrepancies The listed specimens, when analyzed by FISH break-apart probes for the reported gene, were found negative, as the percentage of typical BrAp signals was below the 15% threshold. However, when atypical signals were taken into account, all of these specimens were considered positive for the presence of a gene translocation. These results were consistent with the detection of the reported fusion gene by molecular testing. FISH, fluorescence in situ hybridization; BrAp, break-apart

Case no	Gene (BrAp) tested	Typical BrAp signals (%)	Atypical signals (%)	Molecular test results
1/19	EWSR1	9.7	56	EWSR1-FLI1
199/20	EWSR1	11	11	EWSR1-FLI1
443/17	EWSR1	14.1	14.8	EWSR1-FLI1
150/19	EWSR1	14.6	38.6	EWSR1-FLI1
310/18	FOXO1	6.4	39	PAX3-FOXO1
7/19	FOXO1	10.2	52.4	PAX3-FOXO1
781/21	FOXO1	3.9	45	PAX3-FOXO1

Specifically, in case 1/19, only 9.7% of the cells were found positive for the typical BrAp signal (1F/1Gr/1R), whereas 56% of the cells harbored the atypical signal of one fusion signal and one red signal (1F/1R).

Accordingly, in case 199/20, only 11% of the analyzed cells were positive for typical BrAp signals. However, in another 11% of the analyzed cells, the atypical signal of 1F/1G was observed. When these specimens were tested, either by RT-PCR or NGS, both were found positive for the presence of the EWSR1/FLI1 fusion gene.

Moreover, in two cases (443/17 and 150/19), FISH results were near the cut-off value. Specifically, 14.1% of the cells in the first case and 14.6% in the second case were found positive for the presence of the typical one fusion signal and one BrAp signal (1F/1Gr/1R). However, atypical signals were also observed in both cases (14.8% for 1F/1Gr and 38.6% for 1F/1R, respectively). Both specimens were also analyzed by RT-PCR and were found positive for the presence of the EWSR1 fusion gene with FLI1.

The same pattern of atypical signals was also observed in two cases of alveolar rhabdomyosarcoma. Specifically, in case 310/18, only 6.4% of the analyzed cells were BrAp-positive (1F/1Gr/1R), while 39% of the cells exhibited the presence of one fusion signal and one green signal (1F/1G).

In another specimen, case 7/19, 10.2% of the analyzed cells were positive for the typical BrAp signal (1F/1Gr/1R), while 52.4% of the cells exhibited the atypical 1F/1G signal. Molecular analysis of these specimens by RT-PCR revealed the presence of the chimeric transcript PAX3/FOXO1.

Finally, case 781/21, another alveolar rhabdomyosarcoma specimen, exhibited the same pattern, typical BrAp signals in 3.9% of the analyzed cells and atypical 1F/1G signals in 45%. This specimen was also found positive for the presence of the PAX3/FOXO1 chimeric transcript by NGS.

It should also be noted that atypical signals were detected in specimens positive for the typical BrAp (1F/1Gr/1R) signal, with percentages ranging from 1.1% to 38.6%.

## Discussion

In this study, we retrospectively evaluated the BrAp FISH results of 135 FFPE soft tissue tumor samples in order to highlight the significance of atypical signals and their potential role in STS diagnosis.

As already mentioned, the BrAp FISH approach is currently the most widely used, despite the fact that dual fusion probes are considered superior to BrAp in terms of sensitivity [5].

This is because the BrAp strategy is more feasible when one translocation partner is largely conserved while the other varies, such as in the case of the EWSR1 gene, which has at least 13 known fusion partners.

Accordingly, in our study, BrAp probes were applied for the detection of translocations involving the EWSR1, SS18, FOXO1, and FUS or DDIT3 genes, based on the specialized pathologist’s initial diagnosis. In most cases, the results were clear and readily interpretable, as also reported in previous studies [7].

Nevertheless, in some cases, multiple signals of the tested gene were observed, and in others, atypical signals were noted.

Specifically, as detailed in the Results section, in at least four cases of EWSR1 FISH analysis, there was at least one typical fusion signal per cell along with either a green or red signal, suggesting the loss of one spectrum signal in a large percentage of cells. However, in all four cases of Ewing sarcoma, the EWSR1/FLI1 fusion gene was detected either by RT-PCR or by NGS, leading to a revision of the initial diagnosis to positive.

Interestingly, atypical signals were also observed in two cases of alveolar rhabdomyosarcoma, where approximately 50% of the cells harbored one fusion and one green signal. The presence of the PAX3/FOXO1 fusion gene was confirmed by either NGS or RT-PCR, and the initial diagnoses were accordingly revised.

These findings are in line with previously published data regarding the loss of one signal [8-12].

Notably, one alveolar rhabdomyosarcoma case involving a young boy was of particular interest. The boy was initially diagnosed in 2019 and treated with surgery. As already noted in the Results section, FISH analysis using a BrAp probe revealed a typical BrAp (1F/1Gr/1R) signal in only 10% of cells, whereas atypical one fusion/one green signals were present in approximately 50% of the cells analyzed (case 7/19). The initial diagnosis, which did not take the atypical signals into account, was a false negative. However, molecular testing later confirmed the presence of the PAX3/FOXO1 fusion, implying that the atypical signals did in fact indicate a translocation.

Unfortunately, the boy experienced a relapse two years later and underwent another surgery. Surprisingly, when the remission specimen was sent to our lab and analyzed by FISH using the BrAp probe for FOXO1, the same atypical signal pattern (1F/1Gr) was observed in approximately 45% of the analyzed cells. Considering the molecular findings from the initial specimen, we proceeded to test for the PAX3/FOXO1 fusion again, which was confirmed by NGS.

Furthermore, the second rhabdomyosarcoma case involved a relapse in a six-year-old boy. He had been initially diagnosed with rhabdomyosarcoma four years earlier. Unfortunately, the primary tumor was never tested by FISH and was diagnosed based on PCR findings. Thus, in this study, we report two rhabdomyosarcoma cases with atypical signals (1F/1Gr), both of which experienced a relapse. This observation supports the notion that atypical signals may serve as genetic biomarkers associated with tumor aggressiveness and/or disease progression.

Additionally, these data support findings from a recent study conducted by the Hellenic Group of Sarcomas and Rare Cancers [12], in which our laboratory also participated. That study emphasized the critical role of expert pathologists and molecular testing in achieving accurate STS diagnosis.

To our knowledge, these are the first strong data to suggest that atypical signals detected in FISH analysis should not be dismissed as technical artifacts or tissue fixation-related errors. On the contrary, they are likely due to complex genetic alterations beyond simple translocations [13,14] and may represent tumor-specific biomarkers.

However, a limitation of our study is the relatively small and heterogeneous sample size. Although 135 sarcoma cases were analyzed, certain histologic subtypes may have been underrepresented. This limits the generalizability of our findings and may have reduced our ability to identify additional cases with atypical signals within each subtype. Furthermore, statistical analysis was not feasible due to the small sample size. Nevertheless, the data presented here could inform future research. It would be of particular interest to explore the potential correlation between these complex genetic alterations and disease relapse in retrospective studies with larger, subtype-specific cohorts of STS cases.

## Conclusions

Our study confirms the utility of BrAp dual-color FISH probes in gene fusion testing and complements the previously reported incidence of various atypical BrAp FISH patterns in the routine molecular genetic diagnosis of STSs. Moreover, our data suggest that the so-called “atypical signals” should be taken into account when diagnosing STS. Their presence may be strong evidence of an underlying translocation, and further molecular testing should be considered. Thus, our study underscores the importance of both pathologists and molecular geneticists paying close attention to these observations in the routine diagnostic evaluation of STS tumors.

Finally, these findings indicate that atypical signals may represent an emerging genetic biomarker, which remains to be clarified in future studies.
